# Internet testing for *Chlamydia trachomatis* in England, 2006 to 2010

**DOI:** 10.1186/1471-2458-12-1095

**Published:** 2012-12-19

**Authors:** Sarah C Woodhall, Bersabeh Sile, Alireza Talebi, Anthony Nardone, Paula Baraitser

**Affiliations:** 1 Health Protection Agency, London, UK; 2 Department of Sexual Health and HIV, Kings College Hospital NHS Foundation Trust, London, UK; 3 Sarah Woodhall, Health Protection Agency, HIV & STI Department, 61 Colindale Avenue, London, NW9 5EQ, UK

**Keywords:** Chlamydia trachomatis, Screening, Internet

## Abstract

**Background:**

In recent years there has been interest in websites as a means of increasing access to free chlamydia tests through the National Chlamydia Screening Programme (NCSP) in England. We aimed to describe and evaluate online access to chlamydia testing within the NCSP.

**Methods:**

We analysed NCSP chlamydia testing data (2006–2010) for 15–24 year olds from the 71/95 programme areas in England where site codes were available to identify tests ordered through the internet. The characteristics of people using online testing services in 2010 were compared with those testing in general practice (GP) or community sexual and reproductive health (SRH) services. We evaluated 58 websites offering free chlamydia tests through the NCSP, and 32 offering kits on a commercial basis for signposting to clinical service and health promotion advice offered.

**Results:**

Between 2006 and 2010, 5% of all tests in the included programme areas were accessed through the internet. The number of internet tests increased from 18 (<1% of all tests) in 2006 to 59,750 in 2010 (6% of all NCSP tests). In 2010 the proportion of NCSP tests accessed online by programme area ranged from <1% to 38%. The proportion of tests with a positive result on the internet was higher than tests from general practice and comparable to those from community SRH services (internet 7.6%; GP 5.6%; Community SRH 8.2%). A higher proportion of people accessing online testing were male, aged 20–24 and reported >1 sexual partner in the past year. Provision of sexual health information and appropriate signposting for those in need of clinical services varied between websites. Service provision within the NCSP was fragmented with multiple providers serving specific geographical catchment areas.

**Conclusion:**

Internet testing reaches a population with a relatively high risk of chlamydia infection and appears acceptable to young men, a group that has been difficult to engage with chlamydia testing. In order to maximise the potential benefit of these services, websites should be consistent with national guidelines and adhere to minimum standards for signposting to clinical care and health promotion information. The current system with multiple providers servicing geographically specific catchment areas is contrary to the geographically unrestricted nature of the internet and potentially confusing for clients.

## Background

The English National Chlamydia Screening Programme (NCSP) aims to reduce the prevalence of *Chlamydia trachomatis* infection (‘chlamydia’) through free opportunistic testing and treatment for sexually active under 25 year olds. Tests are available from a wide range of venues including general practice (GP), community pharmacy, community sexual and reproductive health (SRH) services and termination of pregnancy services. The NCSP has driven very large increases in chlamydia testing in England since 2003 with over 2 million tests delivered in 2011 [1].

In recent years there has been interest in websites as a means of increasing access to free chlamydia tests. Website-based testing has been found to be acceptable to young people [2-7] and has been made possible by new tests that can be done on non-invasive samples and increasing access to the internet at home. Seventy seven percent of households in Great Britain have internet access, up from 73 percent in 2010 [8] and 99% of adults aged 16–24 had used the internet in the first quarter of 2012 [9]. Website-based testing offers the advantage of increased convenience and confidentiality, as the service can be accessed 24 hours a day and there is no need to attend a clinic to obtain a test. However, it requires trust that the provider will maintain confidentiality, private access to the internet and privacy to receive postal tests and to take samples. At present the most commonly used model of internet-based chlamydia testing in England is ordering a test on the internet which is delivered to the home where the client takes the sample, posts it to the laboratory and accesses the result via text message. Additional services might include partner notification for those with positive tests delivered by phone and treatment posted home.

In response to the increasingly important role of the internet for chlamydia testing, we analysed NCSP testing data to describe the populations using internet testing and the positivity rates for internet tests in comparison with those from other venues. In addition we assessed testing services against NCSP quality standards for health promotion and signposting (i.e. directing to clinical services where appropriate).

## Methods

### Analysis of internet testing through the NCSP

The Health Protection Agency (HPA) collects individual-level data on all chlamydia tests performed through the NCSP. The HPA has authorisation to use these data for public health purposes (anonymised data are available on request). In 2010 the NCSP was delivered by 95 programme areas and data is available by programme area. Every test performed through the NCSP is assigned a site code which is used to identify the venue testing type. However not all programme areas use codes that can specifically identify tests accessed through the internet (herein referred to as ‘internet tests’), for example, some areas count internet testing within the category ‘remote testing’ which also includes posted invitations to test. We therefore contacted all programme areas to ascertain which codes were used for internet testing between 2006 and 2010. Seventy one of the 95 programme areas (75%) had site codes specifically assigned to internet tests, meaning that these could be identified in the NCSP chlamydia testing data.

For the 71 programme areas with available data, we used NCSP chlamydia testing data to describe the trends in the number and proportion of tests carried out via the internet between 2006 and 2010, and by programme area for 2010. We also undertook a descriptive analysis of the characteristics of people who accessed a test via the internet compared to those who took a test in GP or community sexual and reproductive health (community SRH) services in 2010. Comparative analyses were limited to 2010 in order to explore the most current information on the use of internet testing. We compared the proportions with reported demographic and sexual behavioural variables among those testing in the three settings. The available variables were: age; gender; ethnic group; reporting a new sexual partner in the previous three months and reporting more than one sexual partner in the previous 12 months. We also included an area-level indicator of deprivation (the Index of Multiple Deprivation, IMD [10]). The IMD combines a number of indicators, which cover a range of economic, social and housing issues, into a single deprivation score for each lower super output area (LSOA) in England (an area of average population of 1,500 persons [11]). This allows each area to be ranked relative to one another according to their level of deprivation. IMD scores were assigned according to the individuals’ postcode of residence. Ranks of IMD scores were grouped into quintiles with group 1 representing the 20% most deprived LSOAs in the country and group 5 the 20% least deprived.

### Quality assessment of internet testing sites

A standardised data collection tool was used to collect information on quality indicators relevant to the internet identified from current key NCSP guidelines (Table 1). A simple yes/no scoring system was used to assess whether websites provided health promotion information and appropriate signposting to clinical services. All known websites offering free chlamydia testing through the NCSP as at March 2010 (n = 58) were assessed. A further 32 websites were identified using an internet search engine (Google) that offered internet tests on a commercial basis (for use in England).

**Table 1:** NCSP core requirements and quality criteria for website assessment

**NCSP core requirement**[16]	**Linked quality indicator**	**Clinical/service implications**
Individuals with symptoms should be referred via local care pathways to competent services.	Is specific information provided on the website about where to go if you are symptomatic?	Those who have symptoms may require urgent clinical care and should be signposted appropriately.
Chlamydia screening is an opportunity for sexual health promotion	Is specific information on the website about	Relevant sexual health promotion advice may reduce risks of chlamydia or other STI.
	a) condom use	
	b) contraception	
	c) how to access other STI tests?	
Sexually active individuals aged under 16 can be offered screening if Fraser competent.	Does the site allow you to request a test if aged under 16?	UK guidelines on access to sexual health services for young people specify that the young person’s competence to consent to the service should be assessed by a trained health professional [18].

## Results

**Figure 1:**
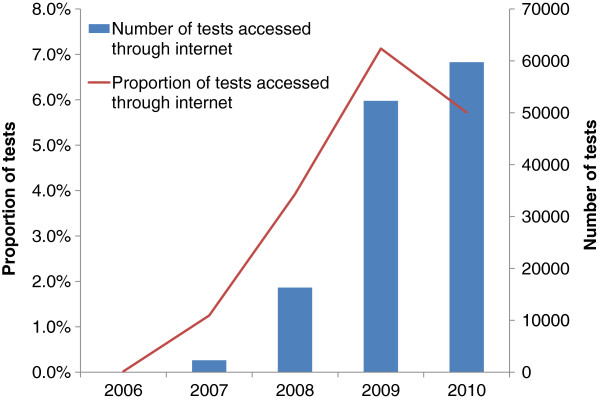
**Number and proportion of NCSP chlamydia tests performed through the internet.** 2006 to 2010 *Includes 71 programme areas (covering 111 PCTs) with specific codes for tests accessed through the internet.

**Figure 2:**
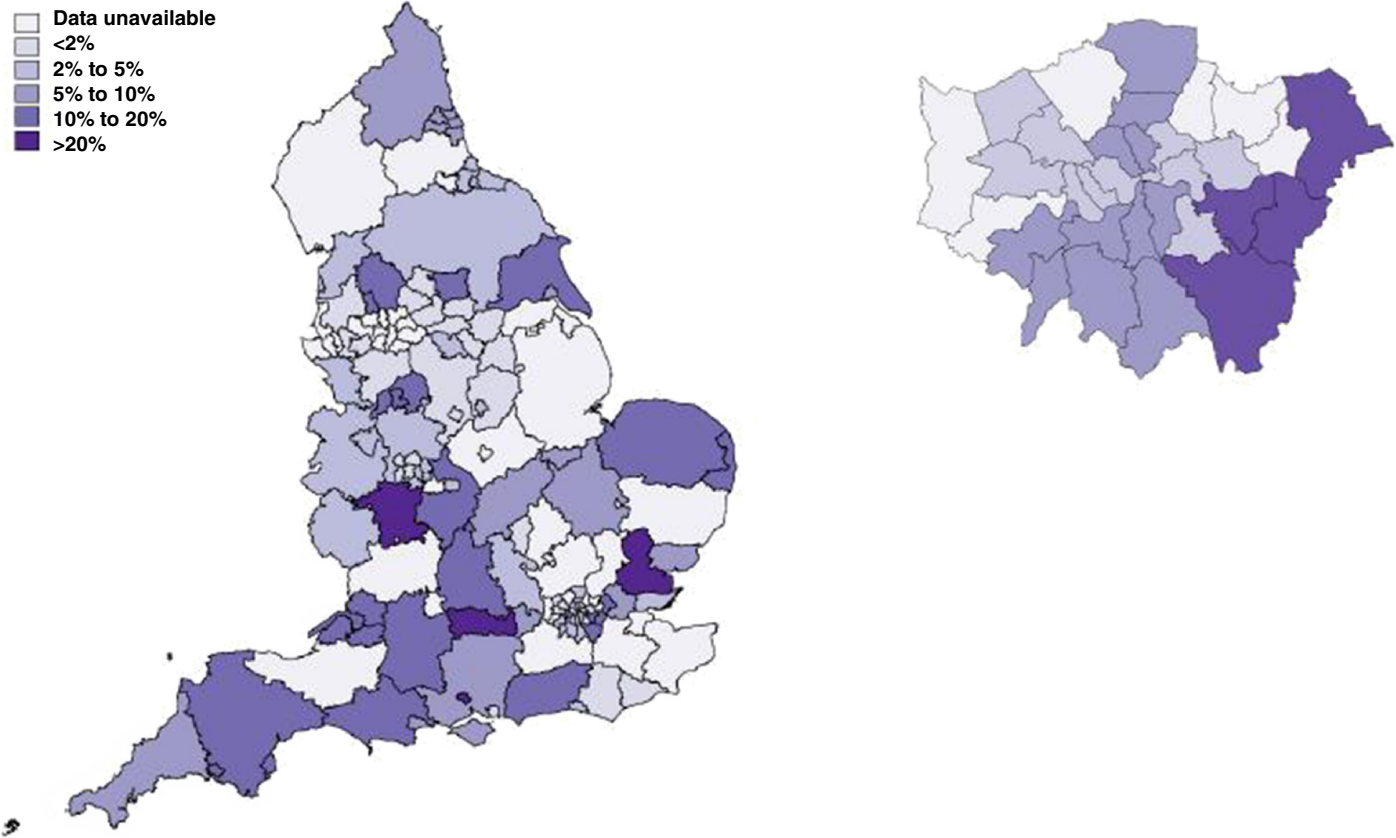
**Proportion of NCSP tests carried out through the internet by programme area, 2010.** (Showing England, with enlargement of the Greater London region). *Includes 71 programme areas (covering 111 PCTs) with specific codes for tests accessed through the internet.

In the 71 programme areas with available data, a total of 2,475,446 tests were reported through the NCSP between 2006 and 2010. Of these, 130,661 (5.3%) were accessed through the internet. The proportion of NCSP tests that were accessed through the internet increased from <0.5% in 2006 to a maximum of 7.1% in 2009 with a slight decline to 5.7% 2010 (Figure 1). The use of internet testing varied by programme area with internet tests contributing between <1% and 38% of tests carried out through the NCSP in 2010 (Figure 2). In programme areas with more than 100 tests contributed by the internet, the proportion of internet tests that were positive ranged from 3% to 19%.

Tables 2 and 3 compare the characteristics of people accessing tests through the internet, GP and community SRH services in 2010. The proportion of internet tests that were positive (8.1% males; 7.3% females) was higher than for tests conducted in GP settings (5.7% males; 5.6% females), and slightly lower, but more comparable to tests performed through community sexual and reproductive health care clinics (9.9% males; 7.7% females) in 2010. Internet tests included higher proportions of men (36%), individuals in the older age group (20–24 years old, 68% males; 65% females) and people of white ethnicity (91% males; 92% females) than tests performed in GP or community SRH services. Higher proportions of women accessing internet testing had a new sexual partner in the previous 3 months (56%) or more than one sexual partner in the past 12 months (59%) than those testing in GP (41% and 38% respectively) or community SRH settings (50%; 49%). The proportion of men reporting these risk behaviours (62%; 68%) was similar to those testing in community SRH settings (65%; 68%), but higher than men tested through a GP (45%; 45%). The proportion of internet tests from each deprivation category (defined by quintile of IMD scores) ranged from 18% to 21% among men and 18% to 22% among women. Both GP and community SRH tests had higher proportions of tests coming from individuals living in the more deprived areas. For example among women, 26% of GP tests and 35% of community SRH tests came from the most deprived areas, compared to 15% and 11% from the least deprived areas.

**Table 2:** Characteristics of 15 – 24 year old males accessing chlamydia tests through the internet, GP and Community SRH services (2010)

	**All NCSP tests**		**Internet**		**GP**		**SRH**	
	**N**	**%**	**N**	**%**	**N**	**%**	**N**	**%**
**Total number of tests**	436,389		21,451		39,432		45,596	
**Test result**								
Positive	18,511	(4.2%)	1,740	(8.1%)	2,251	(5.7%)	4,504	(9.9%)
**Age group**								
15	18,405	(4%)	237	(1%)	901	(2%)	3,215	(7%)
16-19	232,692	(53%)	6,659	(31%)	14,283	(36%)	24,866	(55%)
20-24	185,292	(42%)	14,555	(68%)	24,248	(61%)	17,515	(38%)
**Ethnicity**								
White	235,570	(81%)	17,926	(91%)	20,976	(70%)	26,261	(78%)
Black	19,178	(7%)	499	(3%)	2,784	(9%)	4,002	(12%)
Asian	22,370	(8%)	519	(3%)	4,744	(16%)	1,488	(4%)
Chinese	1,121	(0%)	24	(0%)	335	(1%)	75	(0%)
Other	1,625	(1%)	59	(0%)	298	(1%)	194	(1%)
Mixed	9,667	(3%)	569	(3%)	950	(3%)	1,703	(5%)
Unknown	146,858		1,855		9,345		11,783	
**Reports new sexual partner in past 3 months**								
No	79,607	(43%)	6,537	(38%)	14,518	(55%)	7,913	(35%)
Yes	105,247	(57%)	10,647	(62%)	11,658	(45%)	14,883	(65%)
Unknown	251,535		4,267		13,256		22,800	
**Reports** >**1 sexual partner in past 12 months**								
No	73,104	(43%)	5,216	(32%)	13,830	(55%)	6,764	(32%)
Yes	98,597	(57%)	11,338	(68%)	11,144	(45%)	14,244	(68%)
Unknown	264,688		4,897		14,458		24,588	
**IMD quintile**								
1 (Most deprived)	109,663	(30%)	3,733	(18%)	10,837	(30%)	15,331	(37%)
2	87,123	(24%)	4,276	(21%)	9,525	(27%)	10,053	(24%)
3	69,344	(19%)	4,353	(21%)	6,186	(17%)	6,395	(16%)
4	55,567	(15%)	4,093	(20%)	4,571	(13%)	5,523	(13%)
5 (Least deprived)	45,781	(12%)	4,164	(20%)	4,495	(13%)	3,952	(10%)
Unknown	68,911		832		3,818		4,342	

*Restricted to 71 programme areas (111 PCTs) using specific codes for tests accessed through the internet GP: General practice; SRH: Community sexual and reproductive health service.

**Table 3:** **Characteristics of 15 – 24 year old females accessing chlamydia tests through the internet, GP and Community SRH services (2010**)

	**All NCSP tests**		**Internet**		**GP**		**SRH**	
	**N**	**%**	**N**	**%**	**N**	**%**	**N**	**%**
**Total number of tests**	604,971		38,268		109,187		156,432	
**Test result**								
Positive			2,806	(7.3%)	6,120	(5.6%)	11,971	(8%)
**Age group**								
15	34,668	(6%)	442	(1%)	3,322	(3%)	13,463	(9%)
16-19	305,364	(50%)	13,099	(34%)	41,698	(38%)	82,264	(53%)
20-24	264,939	(44%)	24,727	(65%)	64,167	(59%)	60,705	(39%)
**Ethnicity**								
White	372,334	(84%)	32,604	(92%)	67,934	(82%)	97,153	(81%)
Black	29,111	(7%)	796	(2%)	5,851	(7%)	11,886	(10%)
Asian	19,062	(4%)	747	(2%)	5,464	(7%)	4,127	(3%)
Chinese	2,248	(1%)	74	(0%)	721	(1%)	510	(0%)
Other	2,381	(1%)	91	(0%)	540	(1%)	733	(1%)
Mixed	16,904	(4%)	1,032	(3%)	2,670	(3%)	6,139	(5%)
Unknown	162,931		2,924		26,007		35,884	
**Reports new sexual partner in past 3 months**								
No	178,995	(53%)	13,970	(44%)	43,200	(59%)	45,852	(50%)
Yes	158,528	(47%)	17,525	(56%)	29,819	(41%)	45,577	(50%)
Unknown	267,448		6,773		36,168		65,003	
**Reports** >**1 sexual partner in past 12 months**								
No	169,648	(54%)	12,129	(41%)	42,450	(62%)	42,218	(51%)
Yes	143,135	(46%)	17,756	(59%)	26,486	(38%)	40,362	(49%)
Unknown	292,188		8,383		40,251		73,852	
**IMD quintile**								
1 (Most deprived)	157,847	(29%)	6,662	(18%)	26,319	(27%)	50,020	(35%)
2	131,388	(24%)	7,965	(22%)	24,489	(25%)	35,086	(24%)
3	103,079	(19%)	7,987	(22%)	18,318	(18%)	24,394	(17%)
4	82,400	(15%)	7,341	(20%)	14,620	(15%)	19,514	(14%)
5 (Least deprived)	71,340	(13%)	7,019	(19%)	15,565	(16%)	15,441	(11%)
Unknown	58,917		1,294		9,876		11,977	

*Restricted to 71 programme areas (111 PCTs) using specific codes for tests accessed through the internet GP: General practice; SRH: Community sexual and reproductive health service.

### Assessment of internet testing sites

As at March 2010, access to free chlamydia tests through the internet for 16 to 24 year olds was available for all but one programme area in England. Internet testing through the NCSP was provided by 58 individual websites. Services were provided by NHS or contracted to voluntary sector and private sector providers. In some cases, testing was available through two different websites. Thirteen websites provided a service to more than one programme area; three websites covered 5 or more programme areas one of which offered chlamydia testing to 14 programme areas, and one that provided chlamydia testing in 49 areas.

As shown in Table 4, the provision of additional health promotion information and signposting to related services varied between sites. Most sites provided information on condom use (85%) and where to go if symptomatic (79%), but fewer sites provided information on contraception (33%) or how to access other sexually transmitted infection (STI) tests (47%). The majority of sites (81%) allowed a user to order a test if they were 15. It was established on further discussion with some programme areas that in some cases this request would be followed up by a health professional, but this was not always the case. Furthermore, 32 commercial websites were assessed of which only a third provided health promotion information on condom use and advice for those with symptoms (Table 5). All websites that offered testing through the NCSP provided testing using nucleic acid amplification tests (NAATs). Most of the commercial websites also offered NAATs based on a sample taken at home sent to a testing laboratory, however six offered point of care tests. Currently available point of care tests are known to have low sensitivity and are not recommended [12].

**Table 4:** Assessment of NCSP internet testing websites

	**N**	**%**
Number of websites identified	58	
**Health promotion information**		
Condom use	49	85%
Contraception	19	33%
How to access other STI tests	27	47%
Signposting if symptomatic	46	79%
Allows you to request a test if under 16	47	81%

**Table 5:** Assessment of commercial internet chlamydia testing services

	**N**	**%**
Number of websites identified	32	
**Health promotion information**		
Condom use	10	29%
Contraception	0	0%
How to access other STI tests	20	60%
Signposting if symptomatic	11	32%

## Discussion

The use of the internet for chlamydia screening in England has increased substantially in recent years and is now available free to 16–24 year olds throughout England via the NCSP. Despite this increase, internet tests contribute a small proportion of all tests within the NCSP.

Current delivery is fragmented with a large number of different service providers each serving specific geographical areas. Both the large number of websites, and the variation observed in the proportion of NCSP tests that came from internet testing reflects local commissioning and service delivery arrangements. The uptake of online testing in each area is also likely to vary according to local marketing, although it was not possible to investigate this further in this analysis. The current geographically based model of service delivery is unlikely to be the most efficient use of resources and is at odds with the capacity of internet testing to go beyond geographical boundaries [13].

Research on who accesses internet-based testing has been highlighted as a priority, due to concerns that such services might attract the ‘worried well’ and that testing delivered via the internet could have low positivity rates [14]. Our results do not, however, support these concerns. We found that a high proportion of internet tests resulted in a positive diagnosis, and that people who took an internet test were more likely to report risk behaviours associated with chlamydia infection when compared with general practice and community SRH services. Internet testing is more evenly distributed across socio-economic status, as classified by area level of deprivation, than testing in general practice and community SRH services which both show more testing in more deprived areas. Like the increase in chlamydia testing with age this may reflect differences in access to private internet facilities.

Although the NCSP aims to encourage equal access by men and women, testing rates are consistently higher among women [1]. Our results suggest that internet testing is acceptable to young men and may be a means of increasing their engagement with the NCSP. While there were more internet tests provided by women than men, there is less discrepancy between access to the internet by men and women than in any other testing venue within the NCSP with the exception of testing in military venues. The ratio of male to female tests is similar to that seen in a recent trial of chlamydia screening in the Netherlands, where 31% of the tests were returned by men [15].

Basic quality standards for online NHS chlamydia screening provision are required. NCSP and other national guidance indicate that individuals with symptoms should be advised to see a health professional in case they require urgent treatment. Health promotion information relating to condom use and contraception should be offered in association with any sexual health test [16,17]. This is especially important given our finding that internet tests appear to access a group with a high risk of chlamydia infection.

English law states that those under 16 (the legal age of consent) must be assessed by a health professional prior to sexual health service provision [18,19]. The lack of restriction on use of websites by under 16 year olds contravenes current guidance on sexual health services for young people under the UK legal age of consent [16]. This finding prompted a specific audit in 2010, and a ‘Lessons learned’ communication was issued to all programme areas to remind them of the need for appropriate arrangements for under 16 year olds requesting tests through the internet. The NCSP is currently developing guidance designed specifically for internet testing sites.

Although we used a large individual level dataset, and reviewed all known websites in 2010, there are limitations to our study. We restricted our analysis of the national dataset to areas that used specific coding for internet tests. Our findings may not therefore be representative of all programme areas. However we have no reason to believe that areas without specific codes would be different from those where codes were available. A substantial proportion of tests had missing data on one or more of the sexual behavioural variables (41%). It is feasible therefore that the observed relationship between testing venue and sexual behaviour may have been different for tests where this information was not provided. However the high test positivity in the internet tests is consistent with having a higher numbers of sexual partners. The data presented are based on the service configuration in 2010. It is feasible that the situation we describe may have changed to some extent due to changes both in the organisation in the healthcare sector in England and in the delivery of internet testing services. For example in November 2012, 11 of the programme areas that had previously offered chlamydia testing through the NCSP no longer offered access to free kits through their own website; for six of these, young people were directed to another website that already offered chlamydia tests for several areas of England. The findings of this analysis will be used to assess the potential for local audit on the use and delivery of internet testing. The proportion of tests carried out through the internet will be an overestimate of the proportion of tests carried out overall in the area, as testing reported through the NCSP does not represent all chlamydia tests performed (tests can also be carried out in sexual health clinics and other venues outside of the screening programme). Between April 2010 and March 2011 63% of all chlamydia tests were performed and reported through the NCSP. Our analysis does, however, demonstrate the variation in the use of internet testing, and the key characteristics of those using such a service.

## Conclusions

Internet testing reaches a population with a relatively high risk of chlamydia infection and appears acceptable to young men, a group that has been difficult to engage with chlamydia testing. Internet testing is more evenly distributed across area classified by level of deprivation than testing in general practice and community SRH services which both show more testing in more deprived areas. In order to maximise the potential benefit of these services, websites should be consistent with national guidelines and adhere to minimum standards for signposting to clinical care and health promotion information. The current system with multiple providers servicing geographically specific catchment areas is contrary to the geographically unrestricted nature of the internet and potentially confusing for clients.

## Competing interests

All authors are employed by the Health Protection Agency in England and work with the National Chlamydia Screening Programme.

## Authors' contributions

SW contributed to the design and implementation of the study undertook the analysis and wrote the first draft of the manuscript; BS contributed to the data collection and analysis; AT contributed to the data collection and analysis; AN and PB contributed to the design of the study and interpretation of findings and helped to draft the manuscript. All authors read and approved the final manuscript.

## Pre-publication history

The pre-publication history for this paper can be accessed here:

http://www.biomedcentral.com/1471-2458/12/1095/prepub
